# Improved efficacy of ultrafiltered xylanase–pectinase concoction in biobleaching of plywood waste soda pulp

**DOI:** 10.1007/s13205-017-0614-z

**Published:** 2017-04-07

**Authors:** Divya Sharma, Sharad Agrawal, Ravi Dutt Yadav, Ritu Mahajan

**Affiliations:** 1 Department of Biotechnology, Kurukshetra University, Kurukshetra, Haryana 136119 India; 2 Trident Limited, Mansa Road, Dhaula, Barnala, 148105 Punjab India

**Keywords:** Biobleaching, Kappa number, Soda anthraquinone pulp, Ultrafiltration, Xylanase, Pectinase

## Abstract

The effect of ultrafiltered xylanase–pectinase concoction produced simultaneously by a bacterial isolate using agro-waste-based media was assessed in prebleaching of plywood waste pulp. Ultrafiltered enzymes caused 12.5% reduction in kappa number at reduced enzyme dose of xylanase–pectinase (4.0–0.8 IU) per gram of pulp under optimized conditions at pH 8.5, temperature 55 °C, and treatment period of 2 h. Using this methodology, amount of Cl_2_–ClO_2_ consumption can be reduced up to 30 and 28.86%. Significant improvement in physical and optical properties of pulp was obtained along with an additional reduction in BOD and COD values up to 18.13 and 21.66% using this novel biodelignification approach. This is the first report showing the advantages of using ultrafiltered xylanase–pectinase over crude enzymes in enhancing the bleaching capacity of pulp. This study focussing on the development of good quality paper with less pollution generating strategy will definitely prove a boon for industries.

## Introduction

The evolution of unconventional biotechnological techniques in various processing industries plays a huge role in solving the vital problems of pollution created by the industries. As far as the environment is concerned, paper manufacturing industries are observed to be the worst offenders. Several toxic organo-chlorine compounds are formed during bleaching by the conventional chemical methods, which are found to be bioaccumulating and harmful for the biological systems (Fiedler et al. 1990; Bajpai et al. 2006). These chlorinated organic by-products include mainly dioxins, which cause severe environmental damage, high COD values of water, and ultimately responsible for expensive waste water treatment (Zhang et al. 2008). Although the economic value of a nation is improved by industrialization, the effluents produced by some industries also cause a significant harm to the environment (Hossain and Rao 2014; Raj et al. 2014; Hossain and Ismail 2015). There is massive challenge for pulp and paper mills in handling dangerous pollutants, looking at the environmental load and legal necessities (Kamali and Khodaparsat 2015). To reduce the pollution created by these industries as well as to enhance the quality of paper, microbial enzymes have gained a unique place due to their potential application in paper and pulp industries in an ecofriendly manner.

Lignin, a complex polyphenolic structure, is covalently linked to cell wall polysaccharides as lignin-carbohydrate complexes. Xylanase helps in bleaching by hydrolyzing the xylan from lignin–xylan complex (Yin et al. 2011), thereby decreases the consumption of chlorine and consequently lowers the release of hazardous chemicals in the effluent, hence creating an ecofriendly technology (Beg et al. 2001). Apart from xylanase, pectinase has also been used in pulp and paper industries. During alkaline conditions of pulping, the release of pectin from fiber structure creates pool of anionic trash in the aqueous phase, which is responsible for the high consumption of cationic additives, and ultimately, these anionic-cationic complexes cause blockage of the drainage system. Pectinase helps to degrade the pectin released into the aqueous phase of pulp (Lund et al. 2004). Crude xylanase–pectinase enzymes have been successfully used in biobleaching of plywood industrial waste soda anthraquinone pulp (Agrawal et al. 2016) and in biobleaching of mixed hardwood kraft pulp (Kaur et al. 2010).

In this report, ultrafiltered (UF) enzymes have been applied in biobleaching of soda anthraquinone (SAQ) pulp. The aim of this work is to compare and evaluate the bleaching potential of ultrafiltered xylano-pectinolytic enzymes with crude xylanase–pectinase concoction, on plywood veneer SAQ pulp. No such type of work using ultrafiltered xylanase–pectinase mixture has been reported in the literature until now. This is the first report showing the role of UF xylanase–pectinase synergism in reducing toxic bleaching chemicals consumption and ultimately the cost of waste water treatment, along with the production of superior quality paper with significant improvement in various physical and optical properties of plywood pulp.

## Materials and methods

### Microbial strain and other materials

Production of enzymes was carried out using cellulase free *Bacillus pumilus* AJK strain (MTCC Accession No. 10414). Birchwood xylan was purchased from Sigma-Aldrich, and all other chemicals used were of high purity grade. Agro-residues, such as wheat bran and citrus peel, were purchased from local market.

### Enzyme production, ultrafiltration, and enzyme activity

The xylanase and pectinase enzymes were produced in 250 ml Erlenmeyer flasks containing 50 ml basal medium (g/l: peptone, 5.0; MgSO_4_.7H_2_O, 2.45; pH 7.0) supplemented with 2% wheat bran and 2% citrus peel under submerged fermentation. After autoclaving, inoculation was done with 2% inoculum of 21 h old and incubated at 37 °C for 48 h under continuous shaking conditions. Microfiltration was done using 0.2 µ membrane cartridge to get the clear cell free extract. The clear supernatant was passed through 3 kDa nominal molecular weight cut-off membrane to concentrate the extract tenfold and to remove low-molecular weight impurities. Enzyme activity was determined by measuring the release of reducing sugars after enzymatic reaction using 3,5-dinitro-salicylic acid method (Miller 1959). Assays were performed under similar conditions as described by Kaur et al. (2010).

### Pulp sample and optimization of various reaction parameters using UF enzymes

The biobleaching ability of UF enzymes was determined by incubating unbleached SAQ pulp sample consisting of 90% plywood industrial waste (veneer), 5% bamboo, and 5% hardwood with xylanase–pectinase enzymes under optimized reaction conditions. Pulp consistency used for all the experiments was 10%, and anthraquinone concentration was 0.08%. Reaction parameters for enzymatic bleaching of SAQ pulp were optimized using one variable at a time approach. Different reaction conditions, such as temperature, pH, enzyme dose, and treatment time, were optimized to obtain the best conditions. SAQ pulp (25 g) was treated with xylanase–pectinase preparations under different pH values ranging from 7.5 to 10.0. Similarly, the pulp was treated with different enzyme dosages ranging between 2.0 and 7.0 IU of xylanase and 0.4–1.4 IU of pectinase per gram of pulp for different time periods from 60 to 200 min and in variable temperature range from 45 to 70 °C. Control samples were also run under the same conditions using heat-inactivated enzymes. After washing the pulp samples with water, handsheets were made using the standard TAPPI methods (TAPPI test methods 1996). Kappa number was determined (TAPPI T236) to find the best effective bleaching condition.

### Bleaching steps and analysis of different physical properties of plywood veneer pulp

Bleaching of control, crude enzymes, and UF enzyme-treated pulp samples (under optimum reaction conditions) was carried out using various steps—Chlorination; Alkali extraction; Chlorine dioxide treatment 1; and Chlorine dioxide treatment 2. To evaluate the percent reduction in chlorine consumption in UF enzyme-treated pulps, chlorination was done with different reducing concentrations of chlorine. In the second step, alkali extraction was done by treating the pulp samples with 2.5% NaOH and 0.8% H_2_O_2_ at 80 °C for 2 h. Alkali-treated pulps were washed with water to remove extra alkali present in the pulp, and a known amount of pulp was used to make handsheets for measuring the brightness and the left over pulp was treated with chlorine dioxide, in two steps (1.0 and 0.3% ClO_2_) stages to remove residual lignin at 70 °C for 3 h. Reduction in consumption of chlorine dioxide in both stages was also determined. After all bleaching steps, pulp filtrates were used to determine the BOD and COD values of pulp effluents. Handsheets were prepared and analyzed for different physical properties viz., Pulp freeness (TAPPI Method 227), Brightness (TAPPI Method 452), Breaking length (TAPPI Method 404), Burst factor (TAPPI Method 403), Tear factor (TAPPI Method 496), Viscosity (TAPPI Method 230), and Kappa number (TAPPI Method 236). All experiments were performed in triplicates.

## Results and discussion

### Optimization of enzymatic processes for biobleaching

**Fig. 1:**
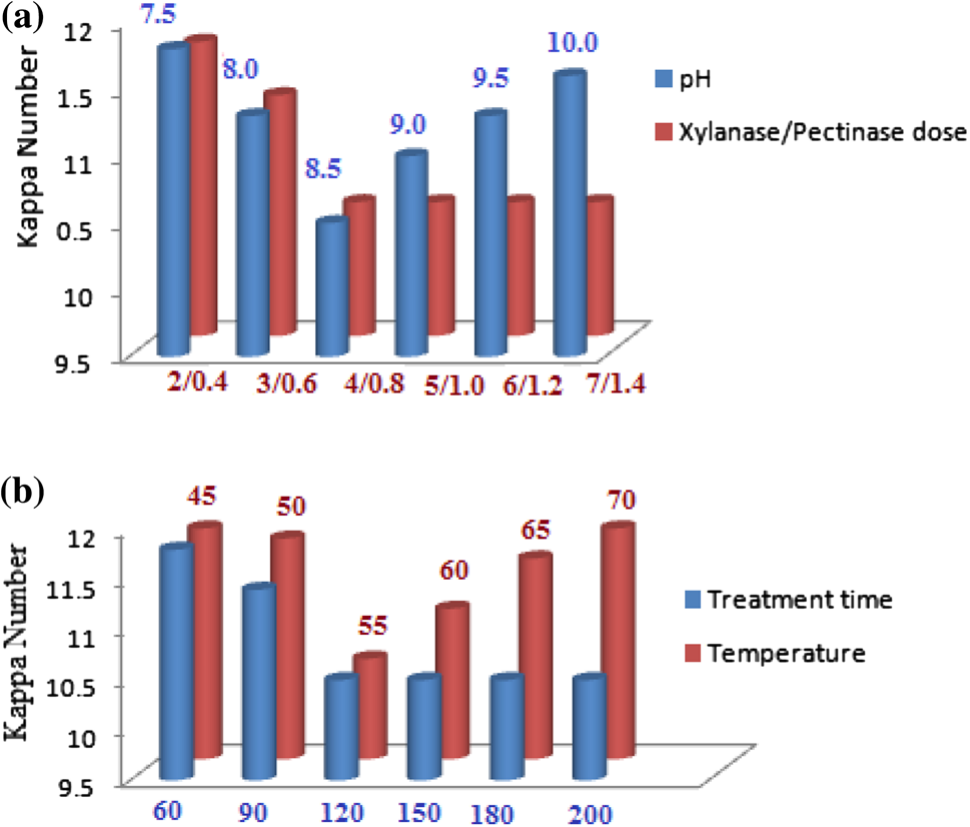
Effect of enzyme dose, pH, treatment time, and temperature on pulp biobleaching. **a** These experiments were performed by treating the pulp at 55 °C for 180 min. For enzyme dose optimization, pH was set at 8.5, and for pH optimization, 4.0 IU of xylanase and 0.8 IU of pectinase dose per g of pulp were used. **b** These experiments were performed at pH 8.5 and enzyme dose of 4 IU of xylanase and 0.8 IU of pectinase per g of pulp. For treatment time optimization, reaction was allowed to proceed at 55 °C, and for temperature determination, treatment time was 120 min

Maximum bleaching of plywood veneer SAQ pulp was observed with enzyme dose of 4.0 IU xylanase and 0.8 IU pectinase per g of pulp at pH 8.5 using UF enzymes (Fig. 1a), whereas 6.0 IU xylanase and 2.4 IU pectinase per g of pulp were found to be optimum for the bleaching of SAQ veneer pulp using crude enzymes at pH 8.5 (Agrawal et al. 2016). Ultrafiltration strategy resulted in the removal of low-molecular weight impurities from the crude xylanase–pectinase preparation, which was interfering with the kinetic efficiency of enzymes. Kaur et al. (2010) reported maximum prebleaching of mixed hardwood and bamboo kraft pulp with enzyme dose of 4.5 IU xylanase and 0.9 IU pectinase at pH 8.5. Xylanase dose of 10 IU/g of pulp at pH 9.0 was found to be optimum for biobleaching of mixed wood kraft pulp (Nagar et al. 2013).

A treatment time of 120 min and temperature 55 °C was found to be optimum for maximum bleaching efficiency of UF enzymes (Fig. 1b). Higher dose of enzymes and longer treatment time did not enhance the biobleaching efficiency. Kaur et al. (2010) reported a treatment time of 180 min at 55 °C for biobleaching of mixed hardwood and bamboo kraft pulp. Treatment time of 2 h at 55 °C was also found optimum in biobleaching of mixed wood kraft pulp (Nagar et al. 2013). Biobleaching of eucalyptus kraft pulp was observed best using enzyme dose of 15 IU xylanase and 20 nkats of laccase per g of pulp (Gupta et al. 2015).

Ultrafiltered enzymatic treatment of SAQ veneer pulp resulted in 12.5% reduction in kappa number over control (non-enzyme treated). Nearly, 9.17% reduction in kappa number over control was obtained by treating SAQ veneer pulp with crude enzymes. Therefore, reduction in kappa number is nearly 3.66% more with UF enzymes in comparison with crude enzymes. The UF preparation enhanced the affinity of enzymes for their selective substrates and resulted in enhancement of delignification efficiency. Kaur et al. (2010) reported 8.5% reduction in kappa number after synergistic action of crude xylanase–pectinase enzymes on kraft pulp. Nearly, 15 and 16.27% reduction in kappa number was observed using combination of xylanase and laccase in biobleaching of eucalyptus kraft pulp and *Hibiscus cinnabinus* SAQ pulp, respectively (Gupta et al. 2015; Andreu and vidal 2014).

### Biobleaching of plywood veneer SAQ pulp

Though the treatment of plywood veneer SAQ pulp with crude xylanase–pectinase synergism resulted in 25 and 23.8% less chlorine and chlorine dioxide consumption, respectively (Agrawal et al. 2016), UF enzyme-treated pulp under optimized conditions, when subjected to chemical bleaching, resulted in 30 and 28.86% reduction in consumption of chlorine and chlorine dioxide, respectively, for obtaining the same % ISO brightness as obtained by the conventional chemical bleaching (Table 1). Biobleaching of mixed hardwood kraft pulp using cellulase free xylanase produced by *Bacillus subtilis* ASH resulted in 28.6% reduction in chlorine consumption (Sanghi et al. 2009). Kaur et al. (2010) reported 25% reduction in chlorine consumption by the synergistic action of xylanase and pectinase enzymes on mixed hardwood kraft pulp. Chlorine consumption was reduced up to 12.5% using xylanase in prebleaching of wheat straw pulp (Walia et al. 2015). Biobleaching of eucalyptus kraft pulp resulted in 20 and 10% reduction in chlorine consumption using xylanase producing *Bacillus halodurans* FNP 135 culture and produced by SmF and SSF, respectively (Sharma et al. 2015).

**Table 1:** Bio-bleaching of soda-AQ pulp using UF xylano-pectinolytic enzymes

Parameters	Control pulp	Crude enzymes treated pulp				UF xylano-pectinolytic enzymes treated pulps			
Step 1		1	2	3	4	1	2	3	4
Chlorination (%)	100	100	80	75	70	100	80	75	70
Cl_2_ added (%)	3.6	3.6	2.88	2.70	2.52	3.6	2.88	2.70	2.52
Cl_2_ consumed (%)	99.2	97	98.5	99	98.6	96.2	98.2	98.9	98.3
Kappa No.	12.0 ± 0.05	9.3 ± 0.05	10.4 ± 0.04	10.6 ± 0.03	10.9 ± 0.02	9.0 ± 0.03	9.7 ± 0.04	10.0 ± 0.03	10.2 ± 0.02
Brightness (ISO %)	35.5 ± 0.15	40.6 ± 0.16	37.2 ± 0.18	36.2 ± 0.16	34.5 ± 0.14	40.9 ± 0.20	37.4 ± 0.15	36.8 ± 0.10	35.4 ± 0.11
Step 2 Alkali added (%)	2.5	2.5	2.5	2.5	2.5	2.5	2.5	2.5	2.5
H_2_O_2_ added (%)	0.80	0.80	0.80	0.80	0.80	0.80	0.80	0.80	0.80
Permanganate number	2.4	1.2	2.5	2.7	3.0	1.4	2.2	2.4	2.6
Brightness (ISO %)	76.5 ± 0.17	78.3 ± 0.18	77.0 ± 0.16	76.7 ± 0.15	76.0 ± 0.13	78.4 ± 0.21	77.6 ± 0.11	77.0 ± 0.12	76.4 ± 0.15
Step 3 D-1 (ClO_2_ added %)	1.0	1.0	0.74	0.675	0.60	1.0	0.74	0.675	0.60
ClO_2_ consumed (%)	95.5	60	81.4	97.6	99.4	56.2	80	88	99.2
Brightness (ISO %)	81.5 ± 0.07	83.4 ± 0.11	82.6 ± 0.13	81.9 ± 0.12	81.3 ± 0.10	84.6 ± 0.09	82.7 ± 0.16	82.4 ± 0.14	82.0 ± 0.15
Step 4 D-2 (ClO_2_ added %)	0.3	0.3	0.3	0.3	0.3	0.3	0.3	0.3	0.3
ClO_2_ consumed (%)	97.5	94	97.4	97.2	97	89.8	95.7	96.8	97.4
Brightness (ISO %)	84.5 ± 0.01	85.5 ± 0.10	84.0 ± 0.14	83.5 ± 0.17	83.0 ± 0.16	86.5 ± 0.10	85.8 ± 0.11	85.1 ± 0.14	84.6 ± 0.12
Step 5 SO_2_ added (%)	2.5	2.5	2.5	2.5	2.5	2.5	2.5	2.5	2.5
Brightness (ISO %)	84.8 ± 0.06	85.8. ± 0.13	85.5 ± 0.17	84.7 ± 0.12	84.2 ± 0.09	86.9 ± 0.11	86.1 ± 0.10	85.5 ± 0.06	84.8 ± 0.10

### Analysis of physical properties of biobleached pulp

Significant improvement in various pulp properties, viz. viscosity, breaking length, burst factor, tear factor, and pulp freeness was observed using UF enzymes. Ultrafiltered enzyme-treated SAQ pulp bleached with 30% less chlorine resulted 11.87, 10.71, 17.42, 14.55, and 5.91% increase in pulp freeness, breaking length, burst factor, tear factor, and viscosity over control, respectively (Table 2). Agrawal et al. (2016) have reported 8.5, 13.4, 10.8, and 4.2% increase in breaking length, burst factor, tear factor, and viscosity, respectively, using crude xylanase–pectinase over control. Higher gains in all physical properties were observed using UF over crude enzymes, where a gain of 6.54% in pulp freeness, 2.1% in breaking length, 3.33% in burst factor, 3.02% in tear factor, 1.54% in viscosity was observed. This indicates that the quality of paper can be greatly improved by including only the fast flow rate, commercially feasible ultrafiltration step. Brightness of ultrafiltered enzymes plus chemically treated pulp was enhanced by about 2.1% in comparison with non-enzyme-treated pulp. Agrawal et al. (2016) have reported nearly 1% increase in brightness in case of crude enzymes plus chemically treated pulp.

**Table 2:** Different physical properties of biobleached soda-AQ pulp

Pulp properties	Control	Crude enzymes treated pulp bleached with 25% less chlorine	UF enzymes treated pulp bleached with 30% less chlorine
Breaking length (m)	5460 ± 6.1	5920 ± 5.1	6045 ± 6.2
Burst factor (kPa m^2^/g)	13.2 ± 0.4	15.0 ± 0.37	15.5 ± 0.25
Tear factor (mN m^2^/g)	53.6 ± 0.43	59.6 ± 0.35	61.4 ± 0.28
Viscosity (cps)	18.6 ± 0.02	19.4 ± 0.07	19.7 ± 0.03
Pulp freeness (^0^SR)	16.0 ± 0.03	16.8 ± 0.08	17.9 ± 0.06

**Fig. 2:**
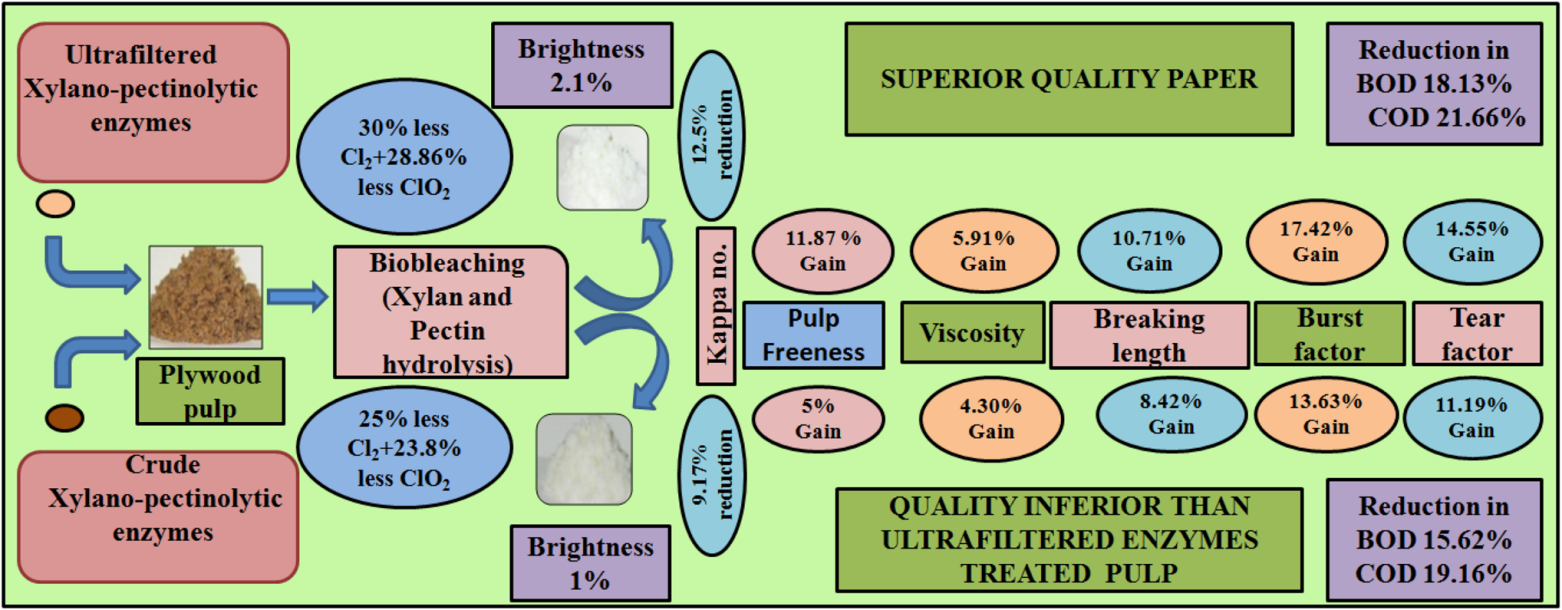
Glimpse of producing superior quality paper from plywood waste using UF xylano-pectinolytic enzymes with reduced generation of pollution

Biobleaching of wheat straw SAQ pulp using xylanase from *Bacillus stearothermophilus* SDX resulted in 4.70, 5.91, 6.96, and 13.15% increase in viscosity, burst factor, breaking length, and tear factor, respectively (Garg et al. 2011). Lin et al. (2013) reported 4.65, 9.3, and 13.95% increase in bursting index after treatment of wheat straw SAQ pulp with pulpzyme HC, recombinant xylanase from *B. halodurans* and commercial xylanase AU-PE89, respectively. Biobleaching of eucalyptus kraft pulp with xylanase producing *B. halodurans* FNP 135 culture and produced by SmF and SSF resulted in 8.6 and 3.3% increase in viscosity, 20.7 and 17.5% increase in tear factor, 13.7 and 12% increase in burst factor, and 8.7 and 6.7% increase in breaking length, respectively (Sharma et al. 2015). Biobleaching of eucalyptus kraft pulp using xylanase and laccase resulted in 13, 49, 6.9, 23, and 11.68% increase in brightness, breaking length, burst factor, tear factor, and viscosity, respectively(Gupta et al. 2015). A glimpse of producing superior quality paper from plywood waste using UF xylano-pectinolytic enzymes with reduced generation of pollution has been completely depicted in Fig. 2.

### Reduction in BOD and COD values of effluents

Effluents released after biobleaching of SAQ pulp with UF enzymes at 30% less chlorination resulted in 2.62 and 9.4 kg/ton BOD and COD values in comparison with control (3.2 and 12.0 kg/ton). Effluents displayed 18.13 and 21.66% reduction in BOD and COD, whereas 15.62 and 19.16% (2.7 and 9.7 kg/ton) reduction in BOD and COD values of effluents was obtained after treating the SAQ plywood veneer pulp with crude enzymes in comparison with control.

## Conclusion

This simple, novel, multi-benefit approach using ultrafiltered xylano-pectinolytic enzymes with low dose can be used to produce superior quality paper in an ecofriendly manner. This technology would also relieve the pressure on paper industries by generating less toxic effluent, which is the main area of concern these days. This new industrially feasible methodology seems to have great scope in paper industry.
